# Molecular Weight Effects of Biscarbazole-Based Hole Transport Polymers on the Performance of Solid-State Dye-Sensitized Solar Cells

**DOI:** 10.3390/nano10122516

**Published:** 2020-12-15

**Authors:** Minseon Kong, Kyeong Seok Kim, Nguyen Van Nga, Yeonju Lee, Yu Seong Jeon, Yunsung Cho, Younghwan Kwon, Yoon Soo Han

**Affiliations:** 1 School of Advanced Materials and Chemical Engineering, Daegu Catholic University, Gyeongbuk 38430, Korea; qewr1666@naver.com (M.K.); kks199@naver.com (K.S.K.); kbg04213@naver.com (Y.L.); db5230@naver.com (Y.S.J.); 2 Department of Chemical Engineering, Daegu University, Gyeongbuk 38435, Korea; vanngatdt@gmail.com; 3 School of Electronic and Electrical Engineering, Daegu Catholic University, Gyeongbuk 38430, Korea; philos@cu.ac.kr

**Keywords:** solid-state dye-sensitized solar cell, biscarbazole, hole-transporting material, hole mobility

## Abstract

The leakage and volatilization of liquid electrolytes limit the commercialization of dye-sensitized solar cells (DSCs). As solid-state (ss) hole-transporting materials, free from leakage and volatilization, biscarbazole-based polymers with different molecular weights (PBCzA-H (21,200 g/mol) and PBCzA-L (2450 g/mol)) were applied in combination with additives to produce ssDSCs. An ssDSC with PBCzA-H showed a better short-circuit current (*J_sc_*), open-circuit voltage (*V_oc_*), and fill factor (*FF*) than a device with PBCzA-L, resulting in 38% higher conversion efficiency. Compared to the PBCzA-L, the PBCzA-H with a higher molecular weight showed faster hole mobility and larger conductivity, leading to elevations in *J_sc_* via rapid hole transport, *V_oc_* via rapid hole extraction, and *FF* via lowered series and elevated shunt resistances. Thus, it is believed that PBCzA-H is a useful candidate for replacing liquid electrolytes.

## 1. Introduction

Dye-sensitized solar cells (DSCs) have garnered attention for their real-world applications such as transparency, multicolor options, easy integration into architecture, and short energy payback time [1,2]. However, the potential problems caused by liquid electrolytes, such as leakage and liquid volatilization, limit their long-term performance and practicality. Solving leakage and volatilization requires replacing the liquid electrolytes in DSCs with a solid-state (ss) hole-transporting material (HTM), and thereby dye desorption can also be prevented. By applying an ssHTM, an ssDSC that is easier to encapsulate and series connect can be realized [3]. More specifically, by merely replacing conventional liquid electrolytes (I^−^/I_3_^−^) with ssHTMs while maintaining the basic structure of conventional DSCs with liquid electrolytes, a liquid-junction-based ssDSC with a configuration of glass/F-doped tin oxide (FTO)/mesoporous TiO_2_:dye/ssHTM/platinized FTO/glass can be embodied.

Recent studies reported the photovoltaic properties of conventional liquid-junction-based ssDSCs [4,5,6,7,8,9]. A 5.4% power conversion efficiency (PCE) was achieved when poly(3,4-ethylenedioxythiophene) was applied as an organic HTM with several additives in a liquid-junction-based ssDSC [4]. Inorganic HTMs, such as CsSnI_2.95_F_0.05_ doped with SnF_2_ [5] and Cs_2_SnI_6_ with additives [6], were also applied, and liquid-junction-based ssDSCs showed PCEs of up to 10.2% and 7.8%, respectively. Moreover, when metal complexes such as a blend of [Cu(tmby)_2_(TFSI)_2_] and [Cu(tmby)_2_(TFSI)] (where “tmby” is 4,4’,6,6’-tetramethyl-2,2’-bipyridine, and TFSI is bis(trifluoromethylsulfonyl)imide) [7], a blend of [Cu(II)(dmp)_2_(TFSI)_2_] and [Cu(I)(dmp)(TFSI)] (where dmp is 2,2’-dimethyl phenanthroline) [8], and a blend of [Co(bpyPY4)](OTf)_2_ and [Co(bpyPY4)](OTf)_3_ (where bpyPY4 is the hexadentate ligand 6,6’-bis(1,1-di(pyridin-2-yl)ethyl)-2,2’-bipyridine and OTf is the trifluoromethanesulfonate anion) [9] were doped with LiTFSI and 4-tert-butylpyridine (TBP) [or 4-(trifluoromethyl)pyridine], the best PCEs of liquid-junction-based ssDSCs were recorded as 11%, 8.2%, and 5.68%, respectively. These liquid-junction-based ssDSCs can be fabricated without a costly and complicate vacuum process (i.e., thermal deposition of metal electrode), compared to ssDSCs with a configuration of glass/FTO/mesoporous TiO_2_:dye/ssHTM/metal electrode (Au or Ag).

Many carbazole derivatives have been applied as HTMs on organic electronics because of their excellent hole-transporting capabilities and chemical stability [3,10,11,12,13]. Carbazole moiety represents a good electron-donating nature due to the nitrogen atoms’ presence, which accounts for its derivatives’ superior hole mobilities. Moreover, a wide variety of functional groups can be introduced to it due to its versatile reactive sites, leading to the facile tuning of electro-optical properties and film-forming capabilities.

In this study, biscarbazole-based hole-transporting polymers with different molecular weights were synthesized using procedures presented in the literature [14,15,16,17], and their molecular orbital energy levels, such as ionization potential, electron affinity, and bandgap energy, were measured. Moreover, we fabricated liquid-junction-based ssDSCs with the biscarbazole-based polymers and investigated the effects of molecular weight on the photovoltaic performance of ssDSCs. A thorough search of the relevant literature yielded no reports of liquid-junction-based ssDSCs with biscarbazole-type polymeric HTMs.

## 2. Materials and Methods

### 2.1. Materials

The following chemicals were purchased from Sigma-Aldrich (St. Louis, MO, USA): *N*-carbazole (≥95%), *N*-2-ethylhexyl bromide (97%), iron (III) chloride (97%), 4-bromophenol (99%), potassium carbonate (≥98%), potassium phosphate (≥98%), copper (II) iodide (98%), trans-1,2-cyclohexanediamine (99%), sodium hydride (60% dispersion in mineral oil), sodium *tert*-butoxide (NaO-*t*-Bu, 97%), tri-*tert*-butylphosphine (P(*t*-Bu)_3_, 90%), tris(dibenaylidene-acetone)dipalladium(0) (Pd_2_(dba)_3_), and *N*-bromosuccinimide (99%). Other chemicals for synthesizing polymeric HTMs were reagent grade and used without further purification. 

Commercial FTO (sheet resistance ~7 Ω/square) glass (TCO22-7), TiO_2_ pastes for the photoelectrode (Ti-nanoxide T/SP) and scattering layer (Ti-nanoxide R/SP), N719 dye (Ruthenizer 535-bisTBA), and hot-melt adhesive (Metlonix 1170-25, DuPont Surlyn) were all purchased from Solaronix (Aubonne, Switzerland). TiCl_4_, LiTFSI, TBP, and 1-methyl-3-propylimidazolium iodide (MPII) were purchased from Sigma-Aldrich (St. Louis, MO, USA). We selected platinum paste (PT-1, acquired from Dyesol-Timo JV, Seoul, Korea) as the Pt counter-electrode source. An indium tin oxide (ITO) with 10Ω/square was purchased from AMG Co., Ltd. (Prides Crossing, MA, USA). A poly(3,4-ethylenedioxythiophene)/poly(styrenesulfonate) (PEDOT:PSS, Baytron P VP Al 4083) was received from H.C. Starck GmbH. All the chemicals for DSC fabrication were used without further purification. 

### 2.2. Synthesis of poly{bis-[6-N-(2-Ethylhexyl)-carbazole-3-yl]-alt-aniline} (PBCzA)

The biscarbazole-based hole-transporting polymers were synthesized using methods presented in the literature [14,15,16,17]. Bis[6-bromo-N-(2-ethylhexyl)-carbazole-3-yl] as a monomer was firstly synthesized [14,15] and then copolymerized with aniline to give PBCzA [16,17]. Detailed procedures, reaction schemes (Schemes S1 and S2), and characterization results (Figures S1–S6) are provided in the Supplementary Materials section.

### 2.3. Fabrication of ssDSCs

Working electrodes with a layer structure of glass/FTO/TiO_2_:N719 dye and counter electrodes with a layer structure of platinized FTO/glass were prepared using the same procedures presented in an earlier work by this study’s researchers [18]. The counter electrodes were placed on the working electrodes and sealed with a 25 μm thick sealing material by annealing for 10 min at 120 °C. 

Synthesized PBCzA (50 mg) was dissolved in 1 mL of chloroform to replace the conventional liquid electrolyte (I^−^/I_3_^−^) with an ssHTM. An additive solution was prepared separately by dissolving LiTFSI (0.066 M, 19 mg), TBP (0.2 M, 27 mg), and MPII (1.0 M, 252 mg) in 1 mL of chloroform [4]. The polymer and additive solutions were mixed with a 3:1 volume ratio before application to the ss hole-transporting layers of the ssDSCs. The resulting mixed solution was then injected into the sealed cells through a predrilled hole on the counter electrodes, and the cells were dried in a vacuum oven for 60 min at 50 °C. This injection and drying process was repeated four times to fill the space between the mesoporous TiO_2_ layer and the platinized FTO layer, thereby fabricating liquid-junction-based ssDSCs with a 25 mm^2^ active area.

### 2.4. Fabrication of Hole-Only Devices

Additive-free hole-only devices were fabricated with a diode configuration of ITO/PEDOT/PSS (30 nm)/PBCzA (100 nm)/Au (70 nm) to estimate the hole mobility of the synthesized PBCzA with molecular weight. The same procedures presented in earlier work [19] were utilized to prepare the hole-only devices, and detailed fabrication conditions are provided in the Supplementary Materials. 

We also fabricated additive-containing hole-only devices with the layer structure of glass/platinized FTO/TiO_2_:N719 dye/HTM with additives/platinized FTO/glass. Both mesoporous and scattering TiO_2_ layers were formed on a counter electrode, and the TiO_2_ was sensitized with N719 dyes. This electrode (glass/platinized FTO/TiO_2_:N719 dye) was sealed with another counter electrode (platinized FTO/glass) using a 25 μm thick sealing material. The mixed polymer and additive solutions were injected into the sealed cell through a predrilled hole on the counter electrode, and the cell was dried in a vacuum oven for 30 min at 50 °C. The injection and drying process was repeated four times to produce additive-containing hole-only devices.

### 2.5. Measurements

The ^1^H and ^13^C NMR spectra were obtained using a Varian Unity Plus 300 spectrometer (Varian Inc, Palo Alto, CA, USA), and chemical shifts were recorded in ppm. UV–visible absorption spectra were recorded on a UV-2100 spectrophotometer (Shimadzu Corporation, Kyoto, Japan), and the photoluminescence (PL) spectra were measured on a RF-5301PC fluorometer (Shimadzu Corporation, Kyoto, Japan). The highest occupied molecular orbital (HOMO) energy level was measured using photoelectron spectroscopy (AC-2, Hitachi High-Tech Corporation, Tokyo, Japan). Molecular weight and molecular weight distributions of polymers were obtained using Waters gel permeation chromatography (515 HPLC pump & 410 differential refractometer, Waters Corp., Milford, MA, USA) equipped with Styragel HR 4E, Styragel HR 3E, and Styragel HR 1E columns while using tetrahydrofuran (THF) as an eluent against polystyrene standards at 40 °C. 

The cross-sectional morphology of the completed ssDSCs was visualized via field-emission scanning electron microscopy (FE-SEM; S-4800, Hitachi High-Technology, Tokyo, Japan). The photocurrent–voltage measurement and electrochemical impedance spectroscopic (EIS) analysis were performed using a CompactStat potentiostat (Ivium Technologies BV, Eindhoven, The Netherlands). A PEC-L01 solar simulator system equipped with a 150 W xenon arc lamp (Peccell Technologies, Inc., Yokohama, Japan) was used as light source. The light intensity was adjusted to 1 sun (100 mW/cm^2^) with a silicon photodiode (model PEC-SI01, Peccell Technologies, Inc., Yokohama, Japan). The dye-adsorbed TiO_2_ films’ active areas were estimated using a digital microscope camera (SZ61, OLYMPUS Corporation, Tokyo, Japan) with image analysis software. All measurements were carried out under ambient conditions at room temperature.

## 3. Results and Discussion

### 3.1. Photophysical and Electrochemical Properties of PBCzAs

**Figure 1:**
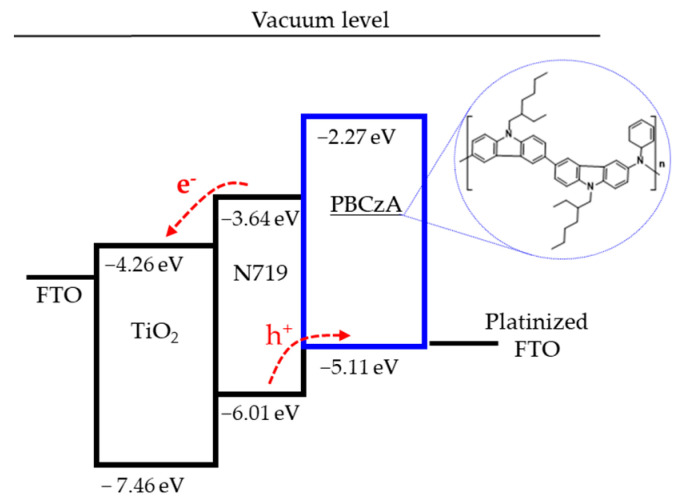
Schematic energy band diagram of solid-state dye-sensitized solar cells (ssDSC) with hole-transporting PCBzA. Inset shows the chemical structure of PBCzA.

Synthesized PBCzA polymer was split into high-molecular-weight (PBCzA-H) and low-molecular-weight (PBCzA-L) fractions via a repetitive reprecipitation procedure. The weight-average molecular weight and molecular weight distribution of polymers were measured at 21,200 g/mol and 2.6 for PBCzA-H and 2450 g/mol and 1.3 for PBCzA-L, respectively (Figure S7). The UV–Vis absorption and PL spectra of PBCzA-H and PBCzA-L in chlorobenzene solutions and films are presented in Figure S8a,b of the Supplementary Materials, respectively. The optical bandgap energy (Eg) of PBCzAs was calculated from the intersection points between the UV–Vis absorption and PL spectra of film-state PBCzAs. In both PBCzA-H and PBCzA-L, the intersection point was observed at 437 nm, corresponding to an Eg of 2.84 eV. As shown in Figure S9, the HOMO energy levels of PBCz-A and PBCz-L were approximately equal within measurement error. We selected −5.11 eV as the HOMO energy of the polymers. Thus, the lowest unoccupied molecular orbital (LUMO) energy levels of PBCzA-H and PBCzA-L were calculated as −2.27 eV. These Eg, HOMO, and LUMO energy levels suggest that the synthesized PBCzAs have potential as a HTM (Figure 1). Molecular weight, molecular weight distribution, and photophysical and electrochemical properties of PBCzAs are summarized in Table 1. 

### 3.2. Photovoltaic Performance of ssDSCs with PBCzA-H and PBCzA-L

**Figure 2:**
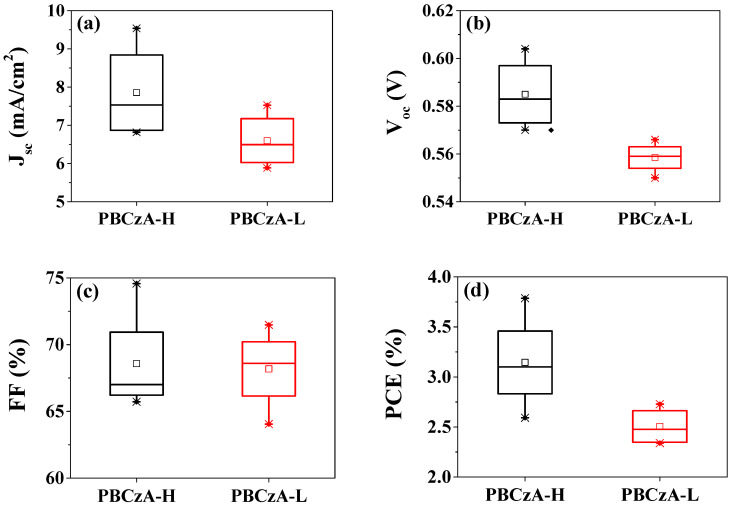
Comparison of photovoltaic parameters with molecular weights of PBCzA: (**a**) *J_sc_*, (**b**) *V_oc_*, (**c**) *FF*, and (**d**) *PCE* of the ssDSCs measured under AM 1.5 (100 mW/cm^2^) illumination. Symbols x, −, □ stand for minimum or maximum, 1% or 99% and averaged values, respectively. The bottom and upper sides of the square indicate lower and upper quartiles, respectively.

We fabricated ssDSCs with PBCzA-H (21,200 g/mol) and PBCZA-L (2450 g/mol) as HTMs and measured their photovoltaic properties to investigate the impact of molecular weight on the device performance. Figure 2 shows the variations in the averaged photovoltaic parameters with the molecular weight of PBCzA. The detailed device performance is compared in Table S1 in the Supplementary Materials. Higher PCEs were achieved in the liquid-junction-based ssDSCs with PBCzA-H compared to the PCEs of devices with PBCzA-L. When the PBCzA-L was applied as a hole-transporting layer combined with additives (LiTFSI, TBP, and MPII), the ssDSCs showed a PCE of 2.51 ± 0.17%. In contrast, an increase in all parameters (i.e., short-circuit current (*J_sc_*), open-circuit voltage (*V_oc_*) and fill factor (*FF*)) resulted in a higher PCE of 3.15 ± 0.43% for the device with PBCzA-H and the additives. 

**Figure 3:**
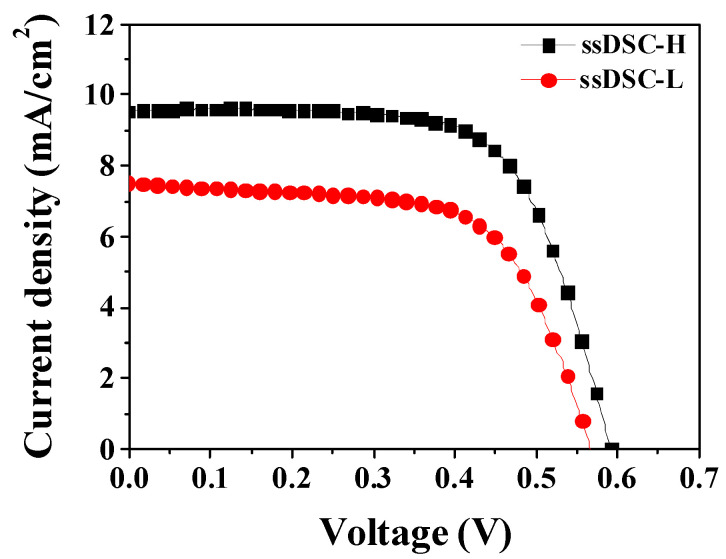
*J*–*V* characteristics of the ssDSC-H and the ssDSC-L.

Revealing the superior PCE’s origins is essential, necessitating a focus on ssDSCs that show the highest PCEs among the four cells. Here, we denote an ssDSC (3.79% of PCE) with PBCzA-H by ssDSC-H and an ssDSC (2.73% of PCE) with PBCzA-L by ssDSC-L, respectively. Figure 3 shows the current density (*J*) and voltage (*V*) curves of the ssDSC-H and ssDSC-L, and the device performance is compared in Table 2. The superior PCE in the ssDSC-H was attributed to better performance in *J_sc_*, *V_oc_*, and *FF* compared to ssDSC-L. We examined the origins of the enhancement in all the photovoltaic parameters, and the analysis results are described in detail in the following sections.

### 3.3. Effects of the Molecular Weight on J_sc_

By utilizing the PBCzA-H as a HTM, the ssDSC-H device’s *J_sc_* value increased by 9.54 mA/cm^2^ compared to ssDSC-L’s 7.53 mA/cm^2^, corresponding to a contribution of approximately 72% to the PCE’s enhancement. The diffusion length (*L_d_*) is the average distance that the excess carriers can cover before they recombine and can be expressed in the following equation:

(1) $$L_{d}=\;\sqrt{2\frac{k_{B}T}{q}\mu \tau }$$

where *μ*, *k_B_*, *T*, *τ*, and *q* are the carrier mobility, the Boltzmann constant, the absolute temperature, the carrier lifetime, and the electron charge, respectively [20,21,22].

**Figure 4:**
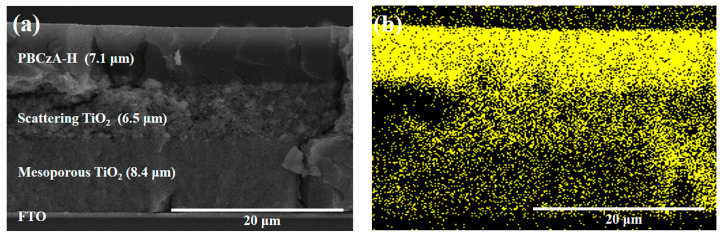
Cross-sectional SEM image (**a**) and EDS mapping (**b**) showing the distribution of carbon in a decapped ssDSC with PBCzA-H (without additives).

In an organic semiconductor, the diffusion length of electrons and holes is about 100 nm, whereas for a single crystalline silicon solar cell, it is typically 100–300 µm [22,23,24]. Thus, the diffusion length (*L_d_*), which is proportional to the square root of the carrier mobility (*μ*), is one of the largest factors affecting *J_sc_* values in ssDSCs. Carrier mobility is particularly critical in liquid-junction-based ssDSCs because the distance between the top of the mesoporous TiO_2_ layer and the Pt counter electrode is about 7.1 μm (Figure 4a). It is well known that polymers poorly penetrate the mesopores of TiO_2_ photoelectrodes, which arises from mismatches between the polymer sizes and the mesopore sizes [4]. Figure 4b shows an EDS mapping image of carbon in a decaped ssDSC with N719 dye-free TiO_2_ and additive-free PBCzA-H layers. As seen in Figure 4b, the polymeric HTM (PBCzA-H) nonuniformly penetrated the mesopores of TiO_2_ photoelectrodes. In other words, the pore filling of the TiO_2_ layer was not complete. EDS mapping images of other elements such as N, Ti, and O are displayed in Figure S10 of the Supplementary Materials. Incomplete pore filling still remained in the case of lower-molecular-weight PBCzA-L, as shown in Figure S11b of the Supplementary Materials.

The additive-free hole-only devices were fabricated using the PBCzA-H and PBCzA-L before their dark current–voltage characteristics were measured to estimate the effects of molecular weight on the hole mobility. Space-charge-limited current (SCLC) measurements have been used to evaluate charge mobility under steady-state currents in organic layers [19,25,26]. Using the SCLC method, we could evaluate the hole mobility of the HTMs (PBCzA-H and PBCzA-L) with different molecular weights. The current density (*J*) in the SCLC model is given in Equation (2):

(2) $$J=\;\frac{9}{8}\varepsilon_{r}\varepsilon_{0}\mu_{h0}\frac{V^{2}}{L^{3}}\exp\left(0.89\sqrt{\frac{V}{E_{0}L}}\right)$$

where *ε_r_* is the dielectric constant (assumed to be three, a typical value for conjugated polymers) of the polymer, *ε_0_* is the permittivity of free space, *μ_h0_* is the zero-field hole mobility, L is the film thickness, E_0_ is the characteristic field, and V is equal to *V_appl_ − (V_r_ + V_bi_)* (*V_appl_*: the applied voltage to the device, *V_r_*: the voltage drop due to series resistance across the electrodes, and *V_bi_*: the built-in voltage).

**Figure 5:**
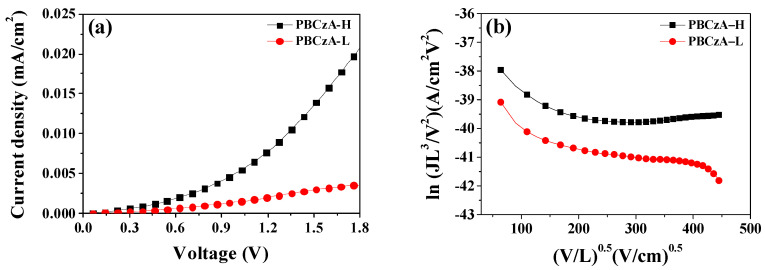
J–V characteristics of the hole-only devices measured in the dark (**a**) and ln (JL^3^/V^2^) versus (V/L)^0.5^ curves (**b**) based on the space-charge-limited current (SCLC) equation.

Figure 5a shows the experimental dark current densities of additive-free hole-only devices with PBCzA-H and PBCzA-L films. The devices’ hole mobility was calculated from Equation (2) using the *J–V* data of the hole-only devices with a configuration of ITO/PEDOT/PSS (30 nm)/PBCzA (100 nm)/Au( 70 nm). This configuration can obstruct the electron injection from the Au electrode due to the large mismatch between the PBCzA’s LUMO energy level (2.27 eV) and the Au cathode’s work function (5.10 eV). In the logarithm of JL^3^/V^2^ versus the square root of V/L (as shown in Figure 5b’s graph), this line’s intercept gives the hole mobility. 

**Figure 6:**
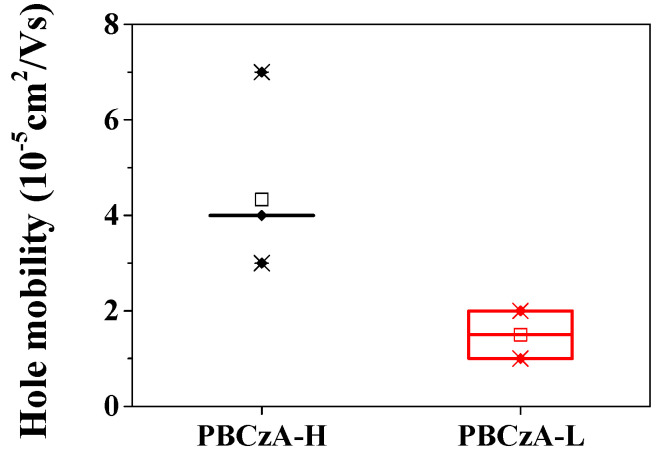
Hole mobilities of PBCzAs calculated using *J*–*V* data of additive-free hole-only devices. Symbols x, −, □ stand for minimum or maximum, 1% or 99% and averaged values, respectively. The bottom and upper sides of the square indicate lower and upper quartiles, respectively.

**Figure 7:**
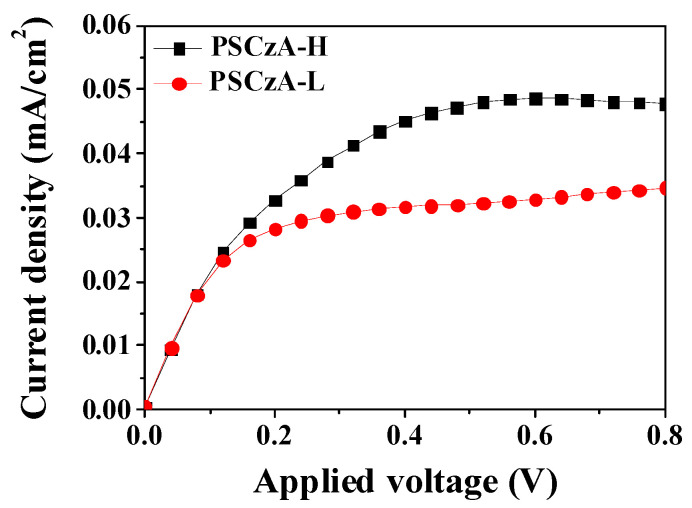
*J*–*V* characteristics of the additive-containing hole-only devices.

The averaged hole mobility of six hole-only devices with PBCzA-L was calculated as 1.50 ± 0.50 × 10^−5^ cm^2^/Vs, whereas the device with PBCzA-H had a value as high as 4.33 ± 1.25 × 10^−5^ cm^2^/Vs (Figure 6). The hole mobility of the additive-free hole-only device with PBCzA-H was about 290% higher than that of the device with PBCzA-L. The increased hole mobility of the device with PBCzA-H was attributed to more effective charge transport through the PBCzA backbone because a longer chain length in PBCzA-H can provide a more effective intramolecular charge transport (i.e., it can decrease the intermolecular charge transport’s frequency) [27,28]. The additive-containing hole-only devices were also fabricated, and their dark current–voltage characteristics were measured to confirm the molecular weight’s effects. As shown in Figure 7, the additive-containing device’s dark currents with PBCzA-H are higher than those with PBCzA-L, which are consistent with the additive-free hole-only devices’ results. This result indicates that the PBCzA-H’s hole mobility is higher than that of the PBCzA-L. 

**Figure 8:**
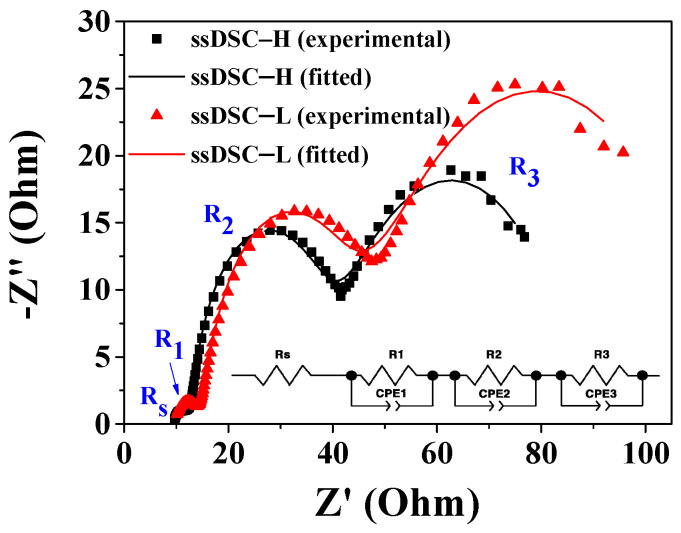
Nyquist plots of EIS spectra for the ssDSCs with PBCzA-H and PBCzA-L, measured at −0.7 V in the dark. Inset shows the equivalent circuit for ssDSC.

Electrical conductivity can estimate carrier hopping in the ssHTM because it is proportional to the product of mobility and carrier concentration. EIS measurements of ssDSCs were performed to calculate the electrical conductivity. Figure 8 shows the Nyquist plots of the EIS spectra for the ssDSCs with PBCzA measured at −0.7 V in the dark, providing the sheet resistance (R_s_) and interface resistances. We observed three distinct semicircles corresponding to the resistances for carrier transport at the Pt/HTM (R_1_) and TiO_2_/N719/HTM (R_2_) interfaces and within the HTM (R_3_) in the ssDSCs. The fitted resistances using a Z-view software are given in Table 3. The conductivity was calculated using *l*/(*A·R_3_*), where *l* is the thickness (around 22 μm) of the HTM between the two FTO glasses, *A* is the photoactive area (0.25 cm^2^) of ssDSCs, and *R_3_* is the fitted resistance determined from the EIS measurements [7]. In the additives’ presence, the electrical conductivity in the ssDSC-H’s PBCz-H (15.88 mS/m) was higher than that of the ssDSC-L’s PBCz-L (10.94 mS/m), indicating a rapid band and hopping transport of holes in the PBCz-H. Thus, a higher *J_sc_* in the ssDSC-H could be attributed to higher hole mobility and electrical conductivity in the PBCzA-H layer than those in the PBCzA-L layer.

### 3.4. Effects of the Molecular Weight on V_oc_

Figure 3 and Table 2 show that the ssDSC-H’s *V_oc_* value (0.590 V) was also higher than that of the ssDSC-L (0.566 V), which accounts for a 13% contribution to the PCE’s improvement. In solar cells based on organic semiconductors, *V_oc_* is generally written as Equation (3):

(3) $$V_{OC}=\;\frac{nk_{B}T}{q}\ln\left(\frac{J_{SC}}{J_{0}}\right)+\;\frac{\Delta E_{DA}}{2q}$$

where *n* is the ideality factor, *J*_0_ is the reverse saturation current density, and Δ*E_DA_* is the activation energy for charge separation at the donor–acceptor interface [29,30,31].

Equation (3) shows that *V_oc_* depends on the charge separation at the donor and acceptor interface (Δ*E_DA_*). Shu et al. reported that high mobility HTMs can extract holes easily from the donor layer, increasing the *V_oc_* [29]. They also investigated the hole mobility effects of various HTMs on the *V_oc_* value in organic solar cells with a configuration of ITO/HTM/donor/acceptor/buffer layer/Al and showed that a HTM with the highest mobility produced the largest *V_oc_* (1.15 V), whereas the other HTMs with lower mobility values exhibited a smaller *V_oc_* value. They attribute the increase in the *V_oc_* to a rapid hole extraction from donor to HTM, leading to effective charge separation (Δ*E_DA_*) [29]. Elumalai et al. also reported that the *V_oc_* in organic solar cells increased linearly with decreasing temperature and then saturated at lower temperatures (around 150 K), which was attributed to the temperature-dependent mobility of organic semiconductors [30]. Conjugated polymers are energetically disordered due to conformational variations [32], which can affect *V_oc_*. Elumalai et al. also found that *V_oc_* decreased with increasing disorder, which was attributed to the disorder-induced carrier traps decreasing the carrier mobility [30]. Thus, the *V_oc_* value is closely related to the carrier mobility, which affects the charge separation.

As mentioned earlier, the hole mobility of the additive-free hole-only device with PBCzA-H was about 290% higher than that of the counterpart with PBCzA-L. Moreover, the dark currents of the additive-containing device with PBCzA-H were higher than those of the device with PBCzA-L. Accordingly, a higher *V_oc_* value in the ssDSC-H was due to an effective charge separation (Δ*E_DA_*), resulting from the higher hole mobility than in the ssDSC-L.

### 3.5. Effects of the Molecular Weight on FF

The ssDSC-H device with PBCzA-H showed a higher *FF* value (67.30%) compared to that of the ssDSC-L device with PBCzA-L (64.05%), corresponding to the 15% increase in PCE. The *FF*, defined as the ratio of the solar cell’s maximum power to the product of *V_oc_* and *J_sc_*, is affected by the series (*R_se_*) and shunt (*R_sh_*) resistances [33,34,35]. A higher *FF* value in solar cells can be achieved by a decrease in *R_se_* and/or an elevation in *R_sh_* [33]. The *R_se_* and *R_sh_* values could be obtained from the slope of the *J–V* curves at *V_oc_* and *J_sc_*, as shown in Equations (4) and (5), respectively [36].

(4) $$R_{se}=\;-{\left(\frac{dJ}{dV}\right)}_{OC}{}^{-1}$$

(5) $$R_{sh}=\;-{\left(\frac{dJ}{dV}\right)}_{SC}{}^{-1}$$

As presented in Table 2, a lower *R_se_* value (11.0 Ωcm^2^) in the ssDSC-H with PBCzA-H was recorded compared to that of the ssDSC-L with PBCzA-L (13.3 Ωcm^2^). Moreover, the *R_sh_* of the ssDSC-H increased by 1238.4 Ωcm^2^ compared to that of the ssDSC-L (1100.1 Ωcm^2^). The *R_se_* value is closely related to solar cells’ internal resistance and can be partially estimated via EIS measurements. As shown in Figure 8 and Table 3, lower resistances at the interface of TiO_2_/N719/HTM (R_2_) and within the HTM (R_3_) were recorded in the ssDSC-H due to PBCzA-H’s higher hole mobility, contributing to the decrease in the ssDSC-H’s *R_se_*. *R_sh_* is influenced by the charge recombination and the leakage current, i.e., the recombination could decrease the *R_sh_* value in the organic solar cells [34]. It is already known that the carrier mobility affects the charge extraction and recombination dynamics, i.e., a more balanced carrier mobility could lead to reduced charge recombination and thereby to a higher *FF* [28,37,38]. One study reported that the electron mobility of inorganic semiconductor TiO_2_ ranged from 0.1 to 4 cm^2^/Vs, which is much higher than the organic HTMs’ hole mobility [39]. In this study, hole mobility measurements showed higher hole mobility in PBCzA-H. These results suggest a more balanced electron and hole mobility in the ssDSC-H than in the ssDSC-L, reducing the charge recombination and increasing the *R_sh_* value in the ssDSC-H. Thus, it can be concluded that the increased *FF* value was attributed to the lowered *R_se_* and the elevated *R_sh_* by the increase of hole mobility in PBCz-H. 

## 4. Conclusions

Polymeric HTMs with both biscarbazole and aniline moieties in the repeating unit were synthesized, and the molecular weight effects on the photovoltaic performance of ssDSCs were investigated. When the lower-molecular-weight PBCzA-L (2450 g/mol) was applied as a hole-transporting layer together with additives, the ssDSCs showed a photovoltaic performance of 6.60 ± 0.63 mA/cm^2^ of *J_sc_*, 0.559 ± 0.006 V of *V_oc_,* and 68.18 ± 2.67% of *FF*, which led to a PCE of 2.51 ± 0.17%. In contrast, in the device with the higher-molecular-weight PBCzA-H (21,200 g/mol), the PCE was increased to 3.15 ± 0.43% (*J_sc_* = 7.86 ± 1.10 mA/cm^2^, *V_oc_* = 0.585 ± 0.013 V, and *FF* = 68.58 ± 3.51%). The enhanced performance was attributed to higher hole mobility and electrical conductivity of PBCzA-H than those of PBCzA-L. Notably, the ssDSC-H showed the highest PCE of 3.79% even though the thickness of the hole-transporting layer (composed of polymeric PBCzA-H and additives) was over 7 μm. This result indicates that a higher-molecular-weight polymeric HTM is a better choice to obtain superior photovoltaic performance in ssDSCs. 

## Figures and Tables

**Table 1:** Summary of molecular weight, photophysical and electrochemical properties of PBCzAs.

HTM	Mw (g/mol)	PDI	λ_max,UV_ (nm)	λ_max,PL_ (nm)	Eg (eV)	HOMO (eV)	LUMO (eV)
PBCzA-H	21,200	2.6	302	450	2.84	−5.11	−2.27
PBCzA-L	2450	1.3	302	448	2.84	−5.11	−2.27

**Table 2:** Performance comparison of ssDSCs showing the highest PCEs.

Devices	HTM	*J_sc_* (mA/cm^2^)	*V_oc_* (mV)	*FF* (%)	*PCE* (%)	*R_se_* (Ωcm^2^)	*R_sh_* (Ωcm^2^)
ssDSC-H	PBCzA-H	9.54	0.590	67.30	3.79	11.0	1238.4
ssDSC-L	PBCzA-L	7.53	0.566	64.05	2.73	13.3	1100.1

**Table 3:** Fitted EIS values for ssDSCs and electrical conductivity of polymeric hole-transporting material (HTMs).

Devices	R_s_ (Ω)	R_1_(Ω)	R_2_(Ω)	R_3_(Ω)	Conductivity(mS/m)
ssDSC-H	9.85	1.65	19.64	55.41	15.88
ssDSC-L	10.08	2.99	20.18	80.46	10.94

## Supplementary Materials

The following are available online at https://www.mdpi.com/2079-4991/10/12/2516/s1, Scheme S1. Synthetic route to bis[6-bromo-N-(2-ethylhexyl)-carbazole-3-yl], Figure S1: ^1^H NMR spectrum of N-(2-ethylhexyl)-carbazole, Figure S2: ^13^C NMR spectrum of N-(2-ethylhexyl)-carbazole, Figure S3: ^1^H NMR spectrum of bis[N-(2-ethylhexyl)-carbazole-3-yl], Figure S4: ^13^C NMR spectrum of bis[N-(2-ethylhexyl)-carbazole-3-yl], Figure S5: ^1^H NMR spectrum of bis[6-bromo-N-(2-ethylhexyl)-carbazole-3-yl], Figure S6: ^13^C NMR spectrum of bis[6-bromo-N-(2-ethylhexyl)-carbazole-3-yl], Scheme S2. Polymerization route to PBCzA, Figure S7: GPC elution profiles for PBCzA-H and PBCzA-L, Figure S8: Absorption and emission spectra of PBCzA in (a) solutions and (b) films, Figure S9: Photoelectron spectroscopy analysis of PBCzA-H and PBCzA-L in a thin film, Table S1: Averages and standard deviations of cell performance, which were measured using four cells with PBCzA layers. Figure S10: Cross-sectional SEM image (a) and EDS mapping images showing the distribution of carbon (b), nitrogen (c), titanium (d), and oxygen (e) in a decapped ssDSC with PBCzA-H (without additives). Figure S11: Cross-sectional SEM image (a) and EDS mapping images showing the distribution of carbon (b), nitrogen (c), titanium (d), and oxygen (e) in a decapped ssDSC with PBCzA-L (without additives). Table S2. Averages and standard deviations of hole mobilities, which were measured using six additive-free hole-only devices.
