# Supplementary material for: Molecular Weight Effects of Biscarbazole-Based Hole Transport Polymers on the Performance of Solid-State Dye-Sensitized Solar Cells
Source: Nanomaterials (Basel). 2020 Dec 15;10(12):2516. doi: 10.3390/nano10122516 (PMC7765262; doi:10.3390/nano10122516)
Supplement: Supplementary file 1 [file nanomaterials-10-02516-s001.pdf]

nanomaterials

Supplementary Materials

Molecular Weight Effects of Biscarbazole-based Hole Transport Polymers on the Performance of Solid-State Dye-Sensitized Solar Cells

Minseon Kong [1,†], Kyeong Seok Kim [1,†], Nguyen Van Nga [2,†], Yeonju Lee [1], Yu-Seong Jeon [1], Yunsung Cho [3], Younghwan Kwon [2,*] and Yoon Soo Han [1,*]

[1] School of Advanced Materials and Chemical Engineering, Daegu Catholic University, Gyeongbuk 38430, Korea; qewr1666@naver.com (M.K.); kks199@naver.com (K.S.K.); kbg04213@naver.com (Y.L.); db5230@naver.com (Y.-S.J.)
[2] Department of Chemical Engineering, Daegu University, Gyeongbuk 38435, Korea; vanngatdt@gmail.com
[3] School of Electronic and Electrical Engineering, Daegu Catholic University, Gyeongbuk 38430, Korea; philos@cu.ac.kr
* Correspondence: y_kwon@daegu.ac.kr (Y.K.); yshancu@cu.ac.kr (Y.S.H.); Tel.: +82-53-850-6561 (Y.K.); +82-53-850-2773 (Y.S.H.); Fax: +82-53-850-6569 (Y.K.); +82-53-359-6662 (Y.S.H.)
† These authors contributed equally to this work.

1. Synthesis of Monomer: bis [6-bromo-N-(2-ethylhexyl)-carbazole-3-yl] (Scheme S1)

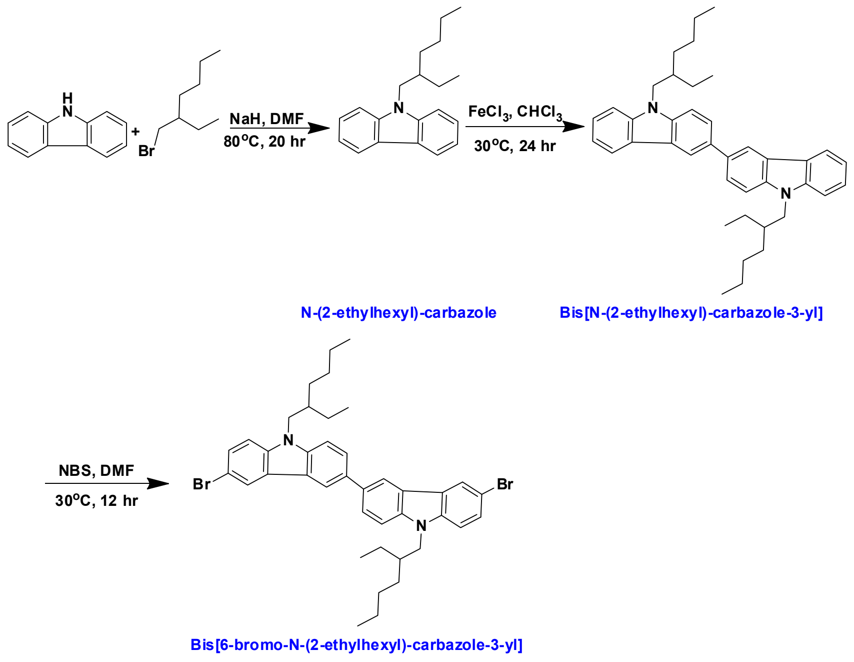

Scheme S1. Synthetic route to bis[6-bromo-N-(2-ethylhexyl)-carbazole-3-yl].

N-(2-ethylhexyl)-carbazole. A mixture of carbazole (10.0 g, 59.81 mmol) and 60% NaH dispersion in mineral oil (3.30 g, 137.50 mmol) was added to 70 mL of DMF, and then the *N*-2-ethylhexyl bromide (13.86 g, 71.77 mmol) was added dropwise. The mixture reacted at 80 °C for 20 h under an N_2 atmosphere. After cooling the mixture to room temperature, the crude product was extracted with diethyl ether/water, dried with magnesium sulfate, and filtered. After evaporating the solvents, the crude product was purified by using column chromatography (eluent: hexane) with silica gel [S1,S2]. Yield 69.0%. ^{1}H NMR (300 MHz, $CDCl_3$) δ 8.17-8.15 (d, 2H, Ar-H), 7.51-7.49 (t, 2H, Ar-H), 7.45-7.29 (d, 2H, Ar-H), 7.26 (t, 2H, Ar-H), 4.19 (d, 2H,-N-CH_2-), 2.13 (s, 1H, -CH-), 1.44-1.35 (m, 8H, -CH-, -

CH_2-), 0.99-0.91 (m, 6H, -CH_3). ^{13}C NMR (75 MHz, $CDCl_3$) δ 141.12, 125.71, 122.99, 120.44, 118.86, 109.13, 47.55, 39.58, 31.22, 29.02, 24.61, 23.26, 14.24, 11.10.

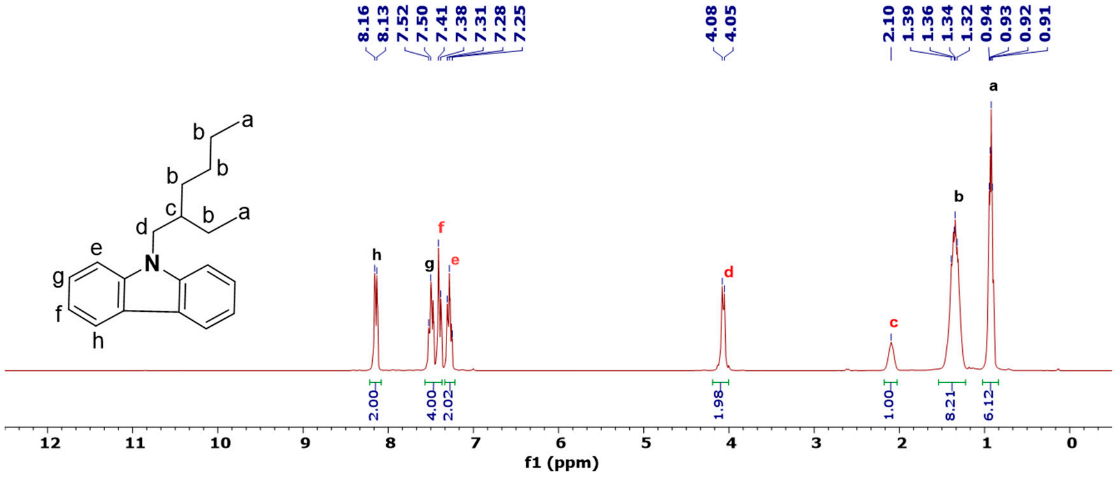

Figure S1. ^{1}H NMR spectrum of N-(2-ethylhexyl)-carbazole.

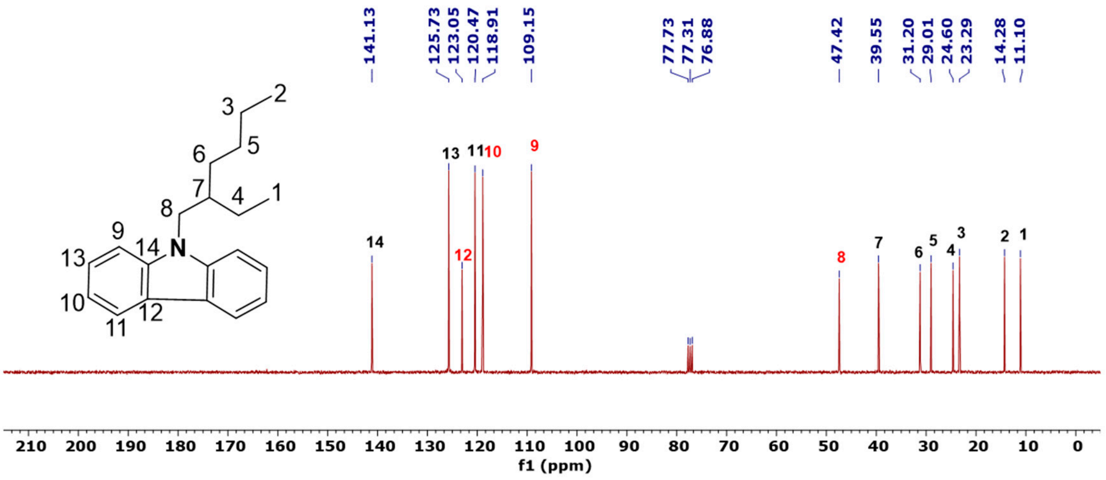

Figure S2. ^{13}C NMR spectrum of N-(2-ethylhexyl)-carbazole.

Bis[N-(2-ethylhexyl)-carbazole-3-yl]. A solution of *N*-(2-ethylhexyl)-carbazole (4.00 g, 14.32 mmol) and chloroform (60 mL) was stirred at room temperature before slowly adding iron (III) chloride (6.65 g, 40.99 mmol). After stirring for 24 h under an N_2 atmosphere, the mixture was extracted with methylene chloride/water, and the organic layer was dried over magnesium sulfate, filtered, and evaporated. The product was purified by column chromatography (eluent: hexane/methylene chloride = 5/1 v/v) with silica gel [S1,S2]. Yield 66.5%. ^{1}H NMR (300 MHz, $CDCl_3$) δ 8.47-8.43 (d, 2H, Ar-H), 8.25-8.22 (d, 2H, Ar-H), 7.87-7.84 (d, 2H, Ar-H), 7.51-7.32 (m, 6H, Ar-H), 7.29-7.27 (m, 2H, Ar-H), 4.16-4.13 (d, 4H, -N-CH_2-), 2.14 (m, 2H,-CH-), 1.42-1.35 (m, 16H, -CH_2-), 0.98-0.91 (m, 12H, -CH_3). ^{13}C NMR (75 MHz, $CDCl_3$) δ 141.53, 140.17, 133.44, 125.75, 125.61, 123.54, 123.22, 120.51, 118.93, 118.86, 109.31, 109.21, 47.58, 39.60, 31.19, 29.01, 24.59, 23.26, 14.25, 11.10.

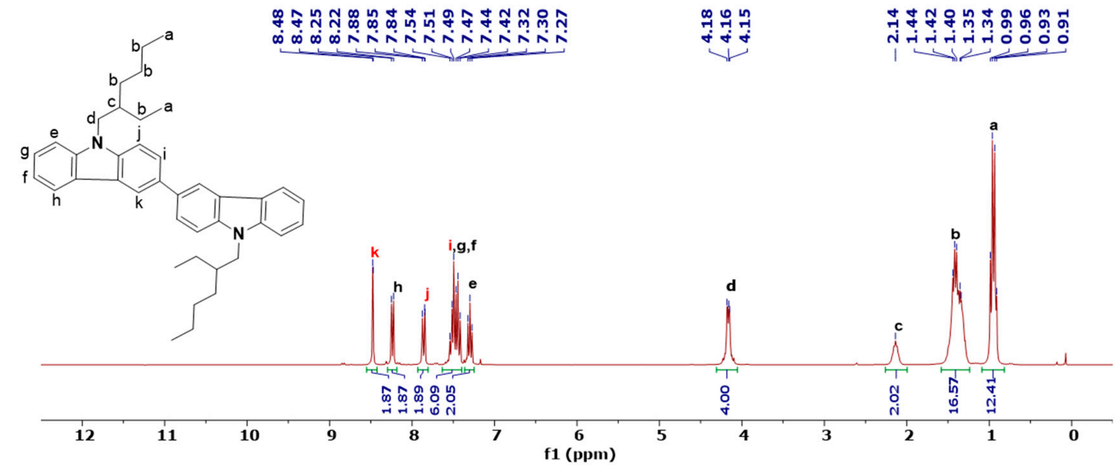

Figure S3. ^1H NMR spectrum of bis[N-(2-ethylhexyl)-carbazole-3-yl].

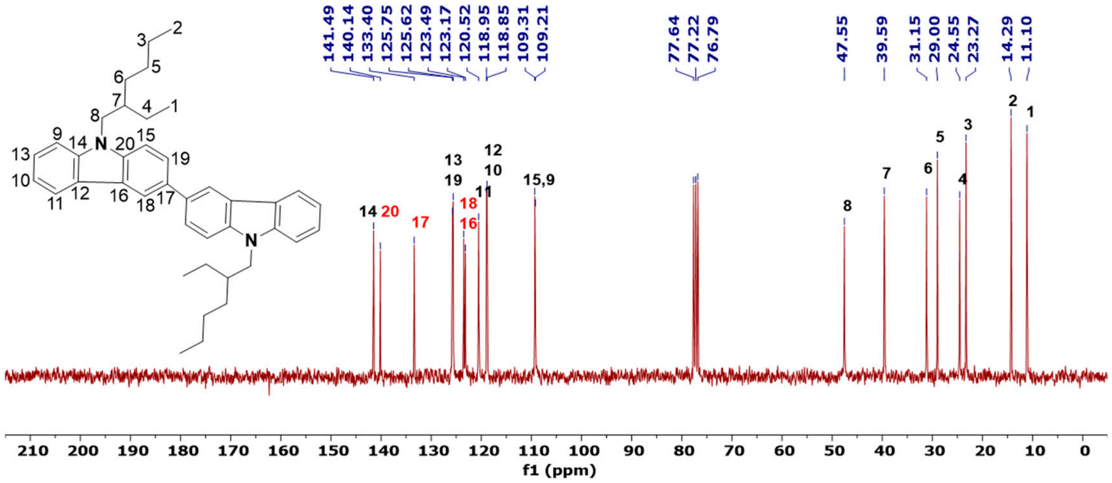

Figure S4. ^{13}C NMR spectrum of bis[N-(2-ethylhexyl)-carbazole-3-yl].

Bis[6-bromo-N-(2-ethylhexyl)-carbazole-3-yl]. A solution of bis[*N*-(2-ethylhexyl)-carbazole-3-yl] (2.50 g, 4.50 mmol) and dimethylformamide (DMF) (25 mL) was stirred in a flask under an N_2 atmosphere before adding N-Bromosuccinimide (1.60 g, 8.98 mmol) at 0˚C. The reaction mixture was allowed to warm in room temperature and reacted for 12 h before it was extracted via diethyl ether/water. The organic layer was dried with magnesium sulfate, filtered, concentrated, then purified by column chromatography (eluent: hexane/methylene chloride = 5/1 v/v) with silica gel [S1,S2]. The synthetic routes to the biscarbazole-based monomer are shown in Scheme 1. Yield 81.3%. ^1H NMR (300 MHz, $CDCl_3$) δ 8.35-8.31(d, 2H, Ar-H), 7.86-7.83 (d, 2H, Ar-H), 7.59-7.56 (d, 2H, Ar-H), 7.50-7.47 (d, 2H, Ar-H), 7.30-7.28 (d, 2H, Ar-H), 4.18-4.16 (d, 4H, -N-CH_2-), 2.11 (s, 2H,-CH-), 1.43-1.34 (m, 16H, -CH_2-), 0.99-0.91 (m, 12H, -CH_3). ^{13}C NMR (75 MHz, $CDCl_3$) δ 140.46, 140.14, 133.51, 128.46, 126.25, 124.87, 123.28, 122.55, 119.06, 111.74, 110.68, 109.65, 47.81, 39.61, 31.19, 28.99, 24.58, 23.24, 14.24, 11.10.

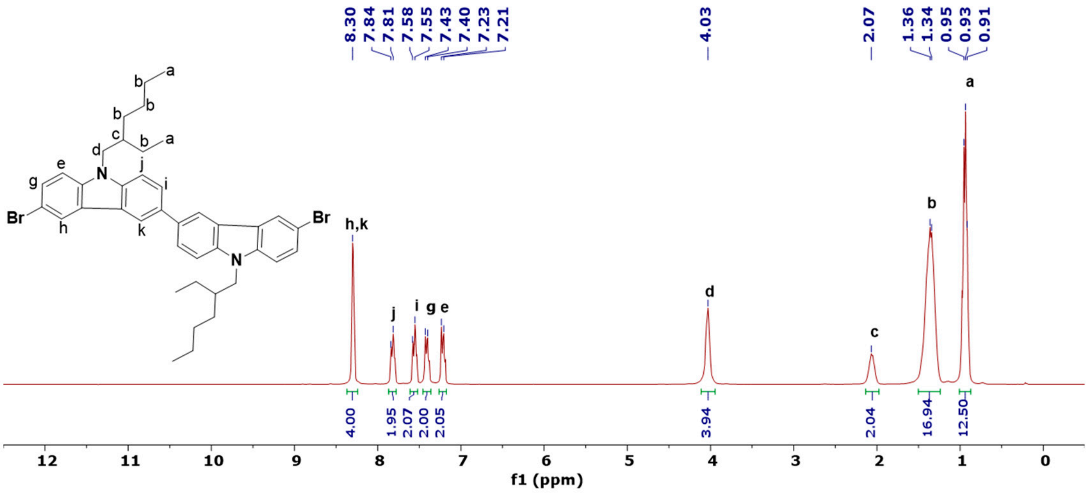

Figure S5. ^{1}H NMR spectrum of bis[6-bromo-N-(2-ethylhexyl)-carbazole-3-yl].

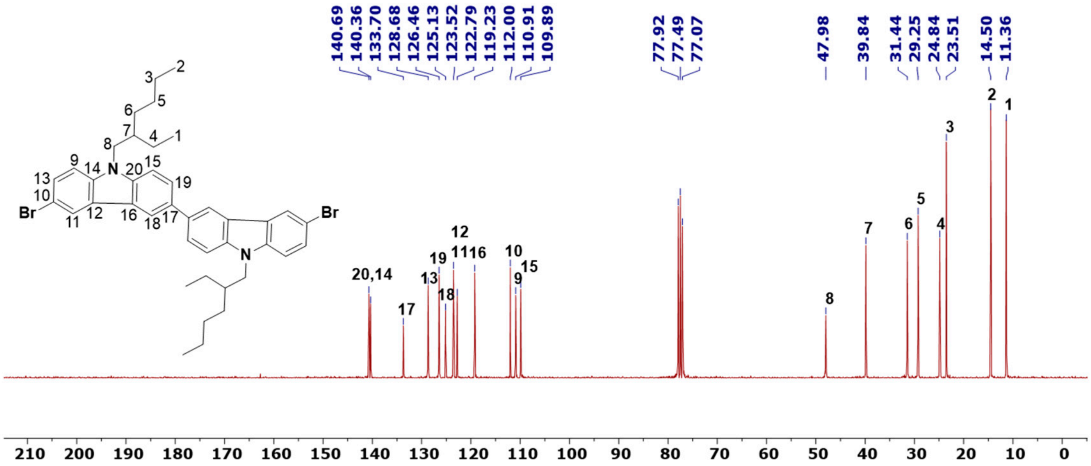

Figure S6. ^{13}C NMR spectrum of bis[6-bromo-N-(2-ethylhexyl)-carbazole-3-yl].

2. Synthesis of Hole-transporting Polymer (PBCzA) (Scheme S2)

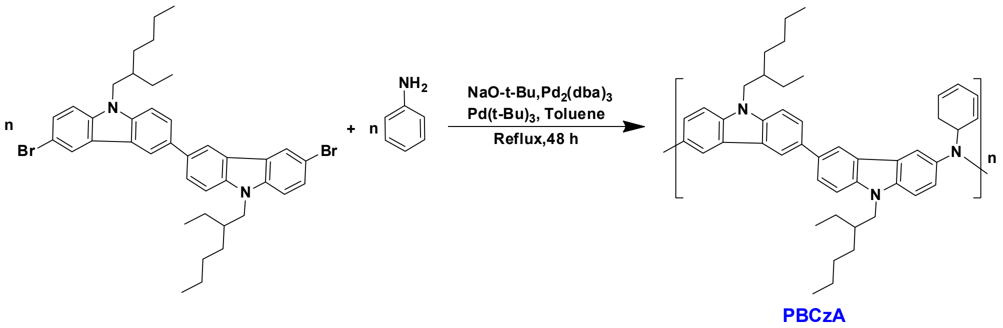

Scheme S2. Polymerization route to PBCzA.

As shown in scheme 2, a mixture of bis[6-bromo-*N*-(2-ethylhexyl)-carbazole-3-yl] (2.29 mmol) and aniline (2.29 mmol) was dissolved in toluene (25 mL). NaO-*t*-Bu (0.6602 g, 6.86 mmol), $Pd_2(dba)_3$ (0.0528 g, 0.06 mmol), and P(*t*-Bu)$_3$ (0.0699 g, 0.35 mmol) were added to the solution at room temperature, and then the reaction mixture was stirred at 100 °C for 48 h. The polymerization was

quenched by adding aqueous ammonia 23% (25 mL), and the polymer was extracted with 25 mL $CHCl_3$. Next, the organic fraction was concentrated and precipitated in MeOH, and then the polymer was filtered and dried in a vacuum [S3,S4]. The polymer was then fractionated in $CHCl_3$/MeOH with a 30/23 (mL/mL) ratio to separate the high molecular weight PBCzA-H and the low molecular weight PBCzA-L.

3. Detailed Conditions for Fabricating Additive-free Hole-only Devices

ITO glasses were cleaned sequentially via ultrasonic treatment in acetone, deionized water, and ethanol and dried in a vacuum oven. Next, ultraviolet-ozone treatment on ITO glasses was conducted for 20 min, and PEDOT/PSS was spin-coated on the ITO glass immediately afterward. The PEDOT:PSS films were then baked on a hot plate for 15 min at 140 °C to give it a thickness of 30 nm. Then, PBCzA solutions were spin-coated on the PEDOT:PSS films and dried to form a 100 nm thick layer. Finally, a 70 nm thick Au electrode was deposited onto the PBCzA layer using a thermal evaporator.

4. Molecular Weight Analysis of Fractionated PBCzAs

4.1. GPC Chromatograms of the Synthesized PBCzAs

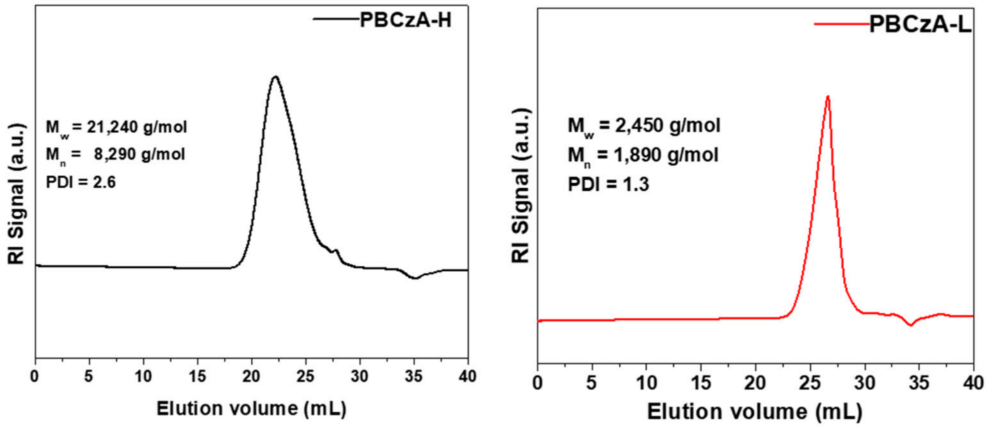

Figure S7. GPC elution profiles for PBCzA-H and PBCzA-L.

5. Optical and Electrochemical Analyses of PBCzAs

5.1. Absorption and Photoluminescence Spectra of the Synthesized PBCzAs

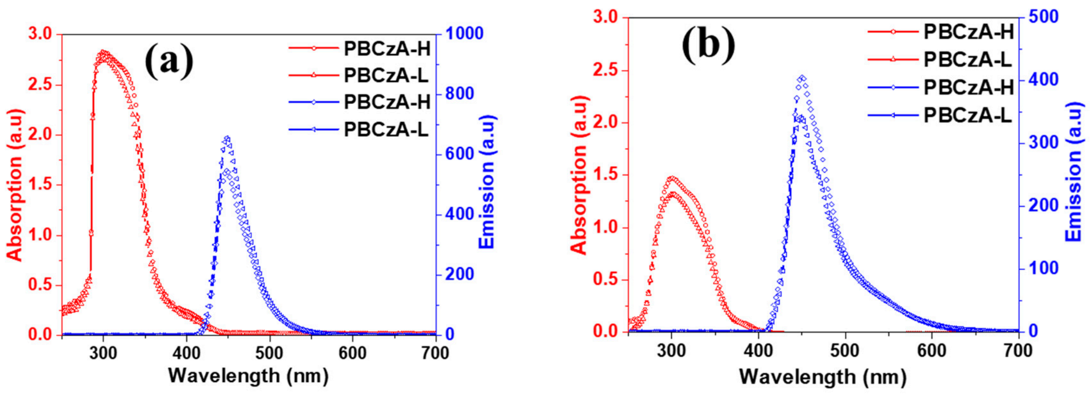

Figure S8. Absorption and emission spectra of PBCzA in (**a**) solutions and (**b**) films.

5.2. Molecular Orbital Energies of the Synthesized PBCzA

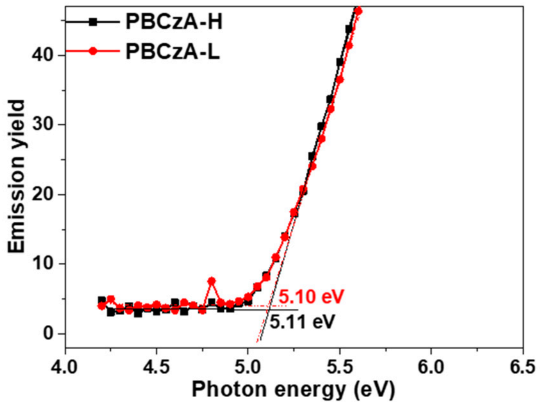

Figure S9. Photoelectron spectroscopy analysis of PBCzA-H and PBCzA-L in a thin film.

6. Photovoltaic Parameters of ssDSCs with PBCzA-H and PBCzA-L

Table S1. Averages and standard deviations of cell performance, which were measured using four cells with PBCzA layers.

HTM	J_{sc} (mA/cm^2)	V_{oc} (mV)	*FF* (%)	*PCE* (%)
PBCzA-H	8.15	0.576	66.71	3.13
	9.54	0.590	67.30	3.79
	6.92	0.570	65.72	2.59
	6.82	0.604	74.58	3.07
	7.86 ± 1.10	0.585 ± 0.013	68.58 ± 3.51	3.15 ± 0.43
PBCzA-L	7.53	0.566	64.05	2.73
	5.89	0.560	71.48	2.36
	6.17	0.550	68.94	2.34
	6.82	0.558	68.26	2.60
	6.60 ± 0.63	0.559 ± 0.006	68.18 ± 2.67	2.51 ± 0.17

7. SEM Image and EDS Mapping of ssDSC with PBCzA

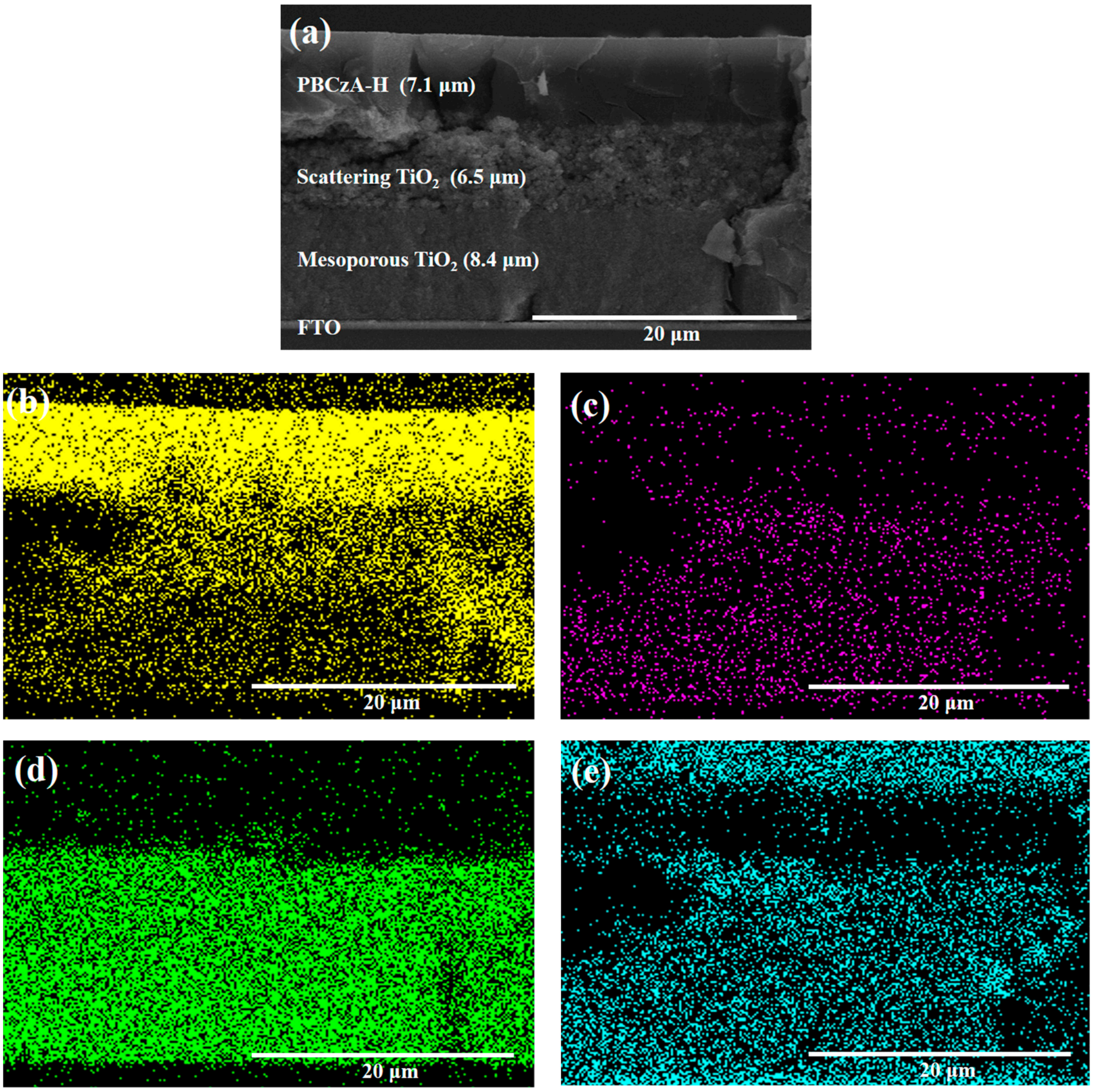

Figure S10. Cross-sectional SEM image (**a**) and EDS mapping images showing the distribution of carbon (**b**), nitrogen (**c**), titanium (**d**), and oxygen (**e**) in a decapped ssDSC with PBCzA-H (without additives).

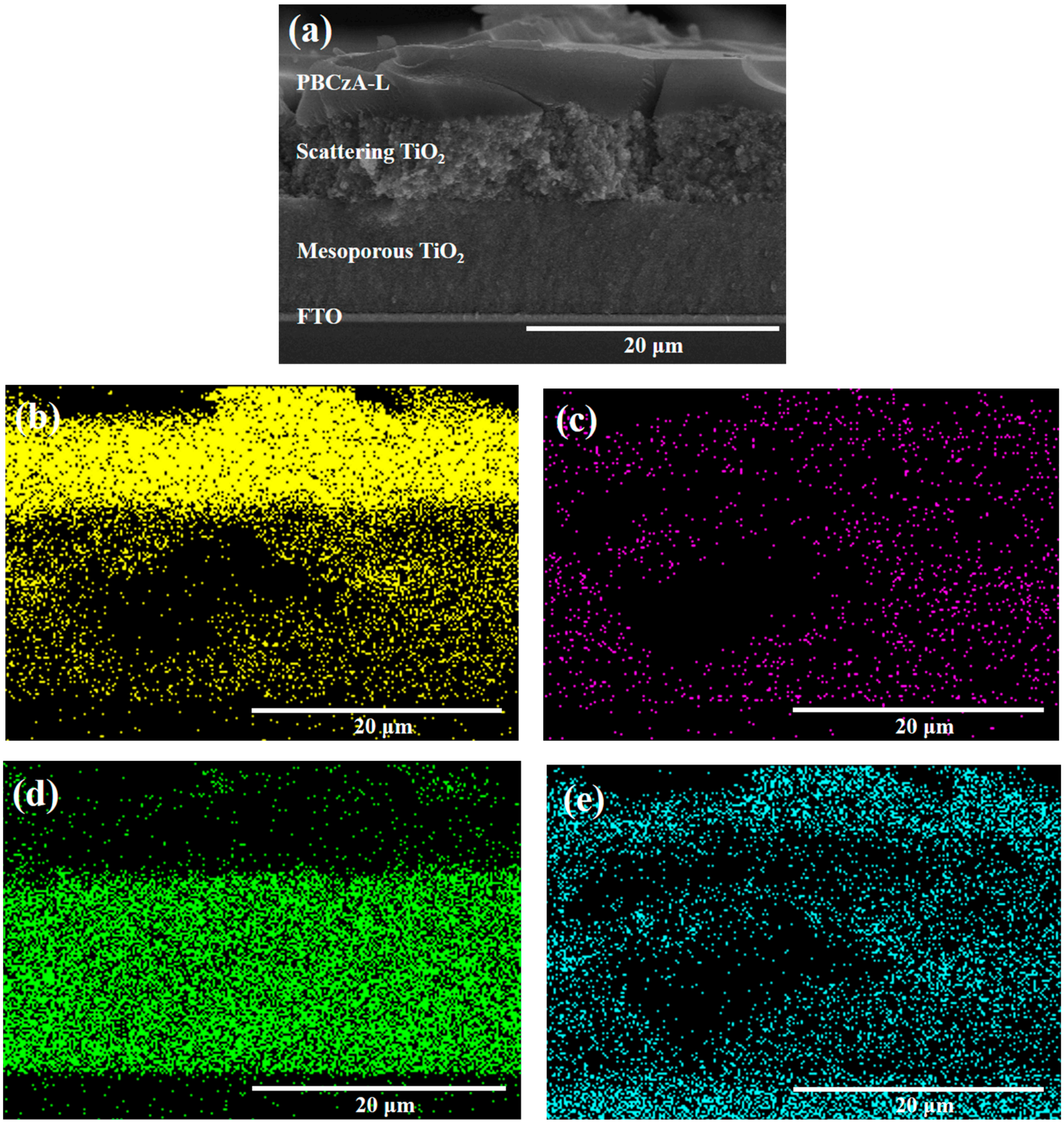

Figure S11. Cross-sectional SEM image (**a**) and EDS mapping images showing the distribution of carbon (**b**), nitrogen (**c**), titanium (**d**), and oxygen (**e**) in a decapped ssDSC with PBCzA-L (without additives).

8. Hole Mobilities of PBCzAs

Table S2. Averages and standard deviations of hole mobilities, which were measured using six additive-free hole-only devices.

Device No.	PBCzA-H (10^{-5} cm^2/Vs)	PBCzA-H (10^{-5} cm^2/Vs)
1	4.0	2.0
2	4.0	1.0
3	4.0	2.0
4	7.0	2.0
5	3.0	1.0
6	4.0	1.0
Average ± standard deviation	4.33 ± 1.25	1.50 ± 0.50

References

[S1] Thaengthong, A-M.; Saengsuwan, S.; Jungsuttiwong, S.; Keawin, T; Sudyoadsuk, T.; Promarak, V. Synthesis and characterization of high T_g carbazole-based amorphous hole-transporting materials for organic light-emitting devices, Tetrahedron Lett. **2011**, *52*, 4749–4752. https://doi.org/10.1016/j.tetlet.2011.07.002

[S2] Bieliauskas, A; Getautis, V; Martynaitis, V; Jankauskas, V; Kamarauskas, E; Krikštolaityt˙e, S; Šačkus, A. Synthesis of electroactive hydrazones derived from carbazolyl-based 2-propenals for optoelectronics, Synth. Met. **2013**, *179*, 27–33. http://dx.doi.org/10.1016/j.synthmet.2013.07.003

[S3] Wang, H.; Ryu, J.-T.; Kwon Y. Carbazole/triarylamine based polymers as a hole injection/transport layer in organic light emitting devices, J. Nanosci. Nanotechnol. **2012**, *12*, 4330-4334. https://doi.org/10.1166/jnn.2012.5897

[S4] Wang, H.; Ryu, J.-T.; Han, Y.S.; Kim, D.-H.; Choi, B.D., Park, L.S.; Kwon Y. Alternating copolymers of *N*-(2-ethylhexyl)-carbazole derivatives with aniline units: synthesis and properties, Mol. Cryst. Liq. Cryst. **2006**, *459*, 85[365]–94[374]. https://doi.org/10.1080/15421400600932272
